# Redefining infantile-onset multisystem phenotypes of coenzyme Q_10_-deficiency in the next-generation sequencing era

**DOI:** 10.20517/jtgg.2020.02

**Published:** 2020-04-23

**Authors:** Andres Berardo, Catarina M. Quinzii

**Affiliations:** Department of Neurology, Columbia University Medical Center, New York, NY 10032, USA

**Keywords:** Coenzyme Q_10_, coenzyme Q_10_ deficiency, coenzyme Q biosynthesis, nephrotic syndrome, cardiopathy, encephalopathy

## Abstract

Primary coenzyme Q_10_ (CoQ_10_) deficiency encompasses a subset of mitochondrial diseases caused by mutations affecting proteins involved in the CoQ_10_ biosynthetic pathway. One of the most frequent clinical syndromes associated with primary CoQ_10_ deficiency is the severe infantile multisystemic form, which, until recently, was underdiagnosed. In the last few years, the availability of genetic screening through whole exome sequencing and whole genome sequencing has enabled molecular diagnosis in a growing number of patients with this syndrome and has revealed new disease phenotypes and molecular defects in CoQ_10_ biosynthetic pathway genes. Early genetic screening can rapidly and non-invasively diagnose primary CoQ_10_ deficiencies. Early diagnosis is particularly important in cases of CoQ_10_ deficient steroid-resistant nephrotic syndrome, which frequently improves with treatment. In contrast, the infantile multisystemic forms of CoQ_10_ deficiency, particularly when manifesting with encephalopathy, present therapeutic challenges, due to poor responses to CoQ_10_ supplementation. Administration of CoQ_10_ biosynthetic intermediate compounds is a promising alternative to CoQ_10_; however, further pre-clinical studies are needed to establish their safety and efficacy, as well as to elucidate the mechanism of actions of the intermediates. Here, we review the molecular defects causes of the multisystemic infantile phenotype of primary CoQ_10_ deficiency, genotype-phenotype correlations, and recent therapeutic advances.

## INTRODUCTION

Coenzyme Q_10_ (ubiquinone; CoQ_10_, EC 206-147-9) is a lipid molecule widely but variably distributed among cellular organelles and tissues. Intracellular CoQ_10_ concentration is highest in the lysosomes and Golgi vesicles, followed by microsomes and mitochondria^[1,2]^. This essential molecule is required for multiple cellular functions and aspects of metabolism, including ATP synthesis via the mitochondrial respiratory chain; antioxidant defenses; regulation of the mitochondrial permeability transition pore; activation of uncoupling proteins; and metabolism of sulfides, proline, arginine, glycine, fatty acids, and pyrimidines^[1,3,4]^. CoQ_10_ contains a long polyisoprenyl tail of ten isoprene units, which positions the molecule in the mid-plane of membrane bilayer, as well as a fully substituted benzoquinone ring that undergoes reversible reduction and oxidation^[3,5]^. The various functions of CoQ_10_ depend on the capacity of the benzoate ring to assume three different redox states: (1) oxidized (ubiquinone); (2) semioxidized (semiubiquinone); and (3) reduced (ubiquinol)^[1–4]^. Although the main ubiquinone antioxidant function is protection against lipid and protein peroxidation, ubiquinol also regenerates other powerful antioxidants, such as α-tocopherol and ascorbate, via electron donation, and recycles them back to their active reduced forms, thereby enhancing activities of other antioxidant defenses^[1–4,6]^.

Among the non-mitochondrial enzymatic systems involved in the continuous regeneration of ubiquinol is selenoprotein thioredoxin reductase (TrxR1), an essential antioxidant enzyme known to reduce many compounds, as well as thioredoxin^[6]^. TrxR1-mediated reduction of CoQ_10_ is dependent on its selenocysteine, which may account for the relationship between levels of ubiquinone and selenium^[7,8]^.

Similar to most other mitochondrial disorders, primary CoQ_10_ deficiency is clinically heterogenous, presenting at different ages of onset, with variable, multiple organs involvement^[9,10]^. In the past, diagnosis of this condition relied only on biochemical assays^[10,11]^. Specifically, low levels of CoQ_10_ in muscle, often, but not always, associated with deficiency of CoQ_10_-dependent respiratory chain enzymes (complexes I + III and II + III) activities^[10]^; however, identification of pathogenic gene variants, wider use of next-generation sequencing, and recognition of characteristic phenotypes have greatly facilitated diagnosis of this condition. For example, the two most frequent and earliest phenotypes associated with CoQ_10_ deficiency, steroid-resistant nephrotic syndrome (SRNS) and cerebellar ataxia, have been linked to specific molecular defects in CoQ_10_ biosynthetic enzymes, and specific COQ genes have been added to targeted diagnostic panels [e.g., *COQ8A*, previously known as *ADCK3*, is included in ataxia gene panels because pathogenic variants in this gene cause autosomal recessive cerebellar ataxia 2 (ARCA2)]^[12,13]^.

In contrast, until very recently, diagnoses of the lethal, infantile or childhood-onset multisystemic forms were reached at late stage of disease or even postmortem, through linkage or homozygous analysis in the family, in conjunction with biochemical diagnosis, and thus fewer patients were reported, compared to the other two phenotypes. However, in the last few years, implementation of next generation sequencing (NGS)-based diagnostics such as whole exome sequencing (WES) and whole genome sequencing (WGS) has caused a dramatic shift in the diagnosis, from a biochemical approach towards a molecular one, of this phenotype too. The unbiased genetic screening approach enables early diagnosis in infants and children with complex multisystemic syndromes, unveiling novel phenotypes, and molecular defects^[14]^; however, it is important to note that some gene variants of uncertain significance have been reported, without the functional studies necessary to prove pathogenicity.

**Figure 1.**
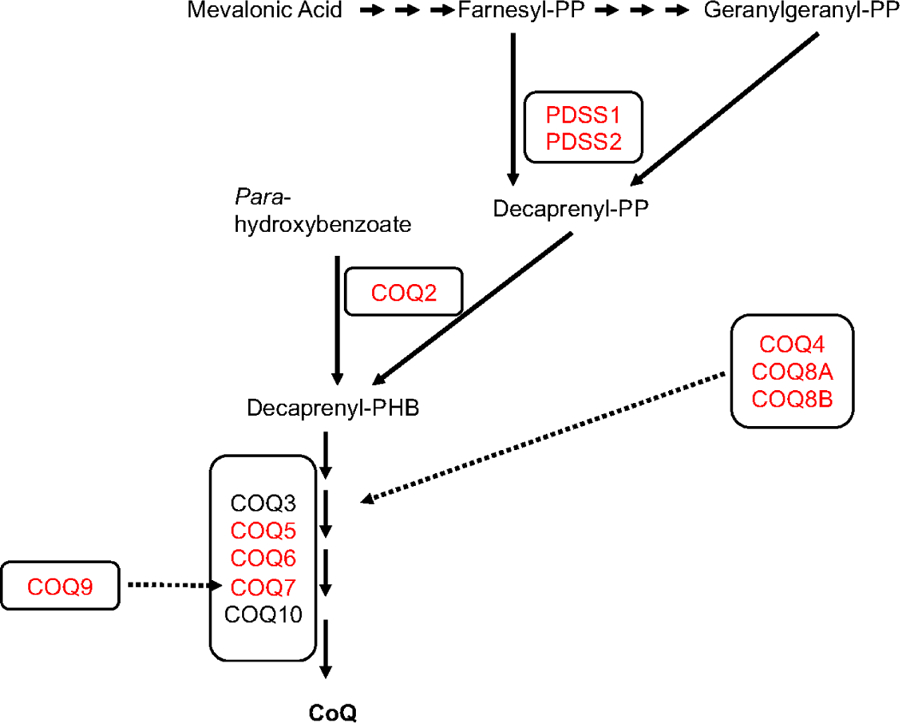
Schematic representation of CoQ_10_ biosynthesis

To date, 10 genes encoding CoQ_10_ biosynthetic proteins have been shown to cause primary CoQ_10_ deficiency: *PDSS1, PDSS2, COQ2, COQ4, COQ5, COQ6, COQ7, COQ8A, COQ8B*, and *COQ9* [Figure 1]. The presentations include: infantile multisystem disease, with variable combinations of encephalopathy, cardiopathy, nephropathy (including SRNS), and cerebellar ataxia; SRNS; and cerebellar ataxia^[9]^. In this review, we focus on the molecular defects in CoQ_10_ biosynthetic genes that cause early-onset multisystemic disease (*PDSS1, PDSS2, COQ2, COQ4, COQ5, COQ7*, and *COQ9*), propose genotype-phenotype correlation, and potential novel therapeutic strategies.

## CLINICAL FEATURES AND MOLECULAR DEFECTS ASSOCIATED WITH EARLY ONSET MULTISYSTEMIC FORMS OF PRIMARY COQ_10_ DEFICIENCY

### *PDSS1* (MIM607429) and *PDSS2* (MIM610564)

Mutations in the gene encoding subunit 1 of the decaprenyl diphosphate synthase [decaprenyl diphosphate synthase subunit 1 (*PDDS1*)], responsible for the synthesis of the decaprenyl tail of CoQ_10_, the first and rate-limiting step of CoQ_10_ biosynthesis [Figure 1]^[5]^, are very rare^[15–21]^. In 2007, Mollet *et al.*^[15]^ reported the first molecularly proven cases: two siblings with CoQ_10_ deficiency manifesting with early-onset deafness, encephaloneuropathy, obesity, livedo reticularis, and valvulopathy, carrying a homozygous missense *PDSS1* pathogenic variant (c.924T>G, p.Asp308Glu).

Another patient, with compound heterozygous for two novel variants (p.Arg221Leufs* and p.Ser370Arg) in *PDSS1* was reported in 2012. The infant presented developmental delay, nephrotic syndrome, and failure to thrive, and died at 16 months of age due to renal failure. Brain MRI showed leukoencephalopathy and brainstem lesions^[16]^.

In 2000, Rötig *et al.*^[17]^ described three siblings with similar symptoms, albeit varying degrees of severity, which included: severe SRNS, neurological impairment (ataxia, dystonia, and amyotrophy), retinitis pigmentosa, sensorineural deafness, and cardiomyopathy. Trans-prenyltransferase deficiency was identified, which was subsequently demonstrated to be due to a homozygous *PDSS2* variant.

In 2006, López *et al.*^[18]^ described an infant with severe Leigh syndrome, nephrotic syndrome, and CoQ_10_ deficiency in muscle and fibroblasts due to compound heterozygous pathogenic variants in *PDSS2* (c.964C>T, p.Glu322* and c.1145C>T, p.Ser382Leu). The patient was hypotonic at birth with rapid evolution of the encephalopathy. At 3 months of age, low dose CoQ_10_ supplementation (50 mg) was initiated, and he developed intractable seizures, progressing to refractory focal status epilepticus, and death at 8 months.

Quinzii and Loos reported another infant, with *PDSS2* pathogenic variants, who presented at age 2 months with severe global developmental delay and failure to thrive. Later evaluations showed bilateral optic atrophy, severe hypotonia, lactic acidosis, renal glomerular dysfunction, Leigh syndrome, and hypertrophy of the left ventricle. At 8 months oral therapy with *L*-carnitine (50 mg/kg/day), CoQ_10_ (10 mg/kg/day), riboflavin (100 mg/day), and thiamine (50 mg/day) was started without clinical response. The proband developed generalized status epilepticus; his neurological status deteriorated and he died at 19 months. Postmortem sequencing identified two novel heterozygous missense mutations: c.590 C>A, p. Ala197Glu and c.932 T>C, p. Phe311Ser^[19]^.

More recently, two novel mutations in *PDSS2* were reported in a 7-month-old infant with nephrotic syndrome, along with encephalomyopathy, hypertrophic cardiomyopathy, deafness, retinitis pigmentosa, and elevated serum lactate level. Clinical exome sequencing revealed a heterozygous missense variant c.485A>G (p.His162Arg) and a heterozygous 2923-bp deletion (c.1042_1148–2816del), which causes a 107-base-long deletion of exon 8. The patient died at 8 months of age, despite CoQ_10_ supplementation (20 mg/kg/day)^[20]^. Pathogenic variants in *PDSS2* were also reported in two patients with isolated SRNS^[21]^.

### *COQ2* (MIM609825)

*COQ2* encodes 4-para-hydroxybenzoate:polyprenyl transferase, the second enzyme in the biosynthetic pathway of CoQ_10_ that condenses the benzoquinone ring with the decaprenyl side chain [Figure 1]^[22]^.

**Figure 2.**
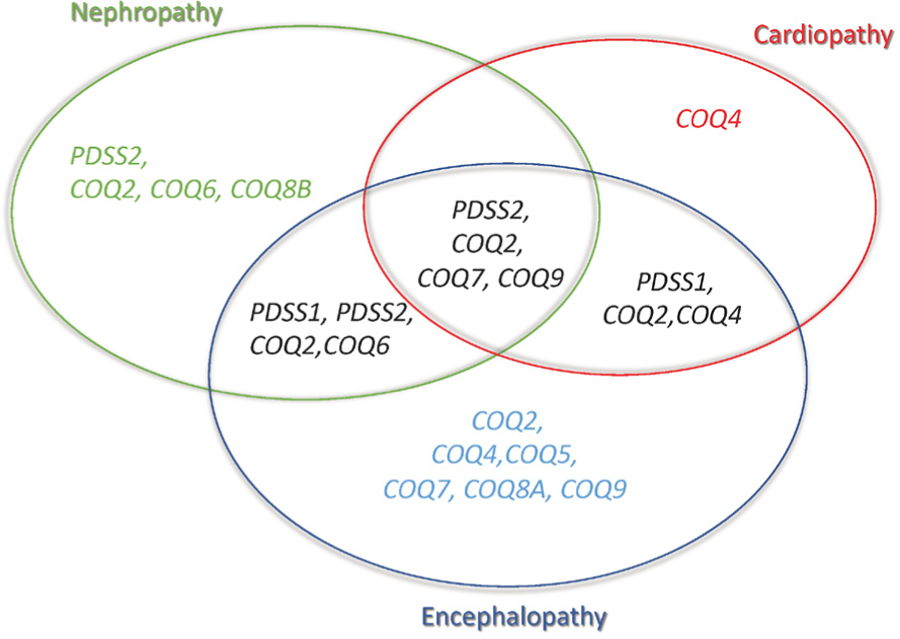
Phenotypes associated with CoQ_10_ biosynthesis defects

Mutations in the *COQ2* gene have been associated with a wide spectrum of phenotypes [Table 1 and Figure 2], which ranges from a rapidly fatal, neonatal-onset, multisystemic disease^[15,23–28]^, to a milder form characterized by SRNS in isolation or associated with encephalopathy^[23,28,29]^. Mutations in COQ2 have also been reported in a patient with Multiple-System Atrophy with retinopathy^[30]^.

The combination of neurological symptoms with SRNS are the hallmark of the neonatal multisystemic presentation of mutations in *COQ2*. SRNS may be the first and predominating feature within the first year of life, followed by later onset of other manifestations such as refractory seizures, hypotonia, psychomotor delay, nystagmus, and optic atrophy^[15,25,26]^. Nevertheless, a few exceptional cases have lacked renal involvement^[26,27]^.

In 2006, Quinzii *et al.*^[24]^ reported the first genetic cause of primary CoQ_10_ deficiency, a homozygous c.890 A>G (p.Tyr297Cys) variant in *COQ2*, in a 33-month-old boy^[23]^. The clinical picture was dominated by nephro-encephalopathy with SRNS (proteinuria 4.3 g/day), psychomotor regression, optic atrophy, tremor, and acute-onset status epilepticus with focal electroencephalogram abnormalities predominantly in the left occipital region. Brain magnetic resonance imaging showed cerebellar atrophy, mild diffuse cerebral atrophy, and stroke-like lesions in the left cingulate cortex and subcortical area. His sister presented only with SRNS at 12 months but was treated before she developed significant neurological manifestations^[23,24]^.

In 2007, Diomedi-Camassei *et al.*^[28]^ described two other patients with early-onset glomerular lesions. The first patient presented with SRNS at the age of 18 months due to collapsing glomerulopathy, with no extrarenal symptoms. He had compound heterozygous *COQ2* variants c.590G>A (p.Arg197His) and c.683A>G (p.Asn228Ser). The second patient presented at 5 days of life with oliguria, with severe extracapillary proliferation on renal biopsy. He rapidly developed end-stage renal disease and died at the age of 6 months after a course complicated by progressive epileptic encephalopathy. He harbored a homozygous c.437G>A (p.Ser146Asn) variant. In 2018, Eroglu *et al.*^[29]^ reported four patients from two different families with SRNS and three with insulin dependent neonatal diabetes were described. Despite initial response to CoQ_10_ supplementation in three, all patients developed neurological features, including intractable seizures that did not improve with oral CoQ_10_ treatment.

In contrast to the original cases with *COQ2* defects and encephalonephropathies, Desbats *et al.*^[25]^ described a neonatal case with severe lactic acidosis, proteinuria, dicarboxylic aciduria, hepatic insufficiency, hypokinetic, and dilated left ventricle on echocardiography, although without clinical signs of cardiomyopathy, who died within the first 24 h of life. Scalais *et al.*^[27]^ described a patient without renal involvement, who presented at 3 weeks of age with myoclonic epilepsy and hypertrophic cardiomyopathy. Serial brain MRIs performed at 4 months showed bilateral and symmetrical increased signal intensities within the posterior putamen and temporal areas and in the rolandic and parasagittal cerebral regions as well as cerebral atrophy and increased CSF lactate. Jakobs *et al.*^[26]^ described dizygotic twins from consanguineous Turkish parents born prematurely who died at the ages of five and 6 months, respectively, after fluctuating disease courses with apneas, seizures, feeding problems, and generalized edema. Again, in these patients, there was no evidence of renal involvement. The patients carried a novel homozygous mutation in *COQ2* (c.905C>T, p.Ala302Val).

### *COQ4* (MIM 616227)

*COQ4* is responsible for the stabilization of CoQ multienzyme biosynthetic supercomplex [Figure 1]^[31]^. Mutations in *COQ4* have emerged lately as common causes of primary CoQ_10_ deficiency manifesting with a variety of phenotypes [Table 1 and Figure 2], dominated by cardiopathy and/or encephalopmyopathy, without renal involvement^[32–40]^.

The initial evidence of *COQ4* dysfunction as cause of encephalomyopathy was the report of Salviati *et al.*^[32]^, who, in 2012, reported a 3.9-Mb deletion of chromosome 9q34.13 encompassing COQ4 in a 3-year-old boy with mental retardation, encephalomyopathy, and dysmorphic features who responded to CoQ_10_ supplementation (30 mg/kg per day of ubiquinone).

In 2015, the first five patients with point mutations in *COQ4* were described. Four of them had prenatal or perinatal onset with early fatal outcome. Two unrelated individuals presented with severe hypotonia, bradycardia, respiratory insufficiency, and heart failure. Two sisters showed antenatal cerebellar hypoplasia, neonatal respiratory-distress syndrome, and epileptic encephalopathy. Only one patient had a gradually progressive condition characterized by spastic ataxic gait and seizures. Except for the solitary patient with the progressive condition, CoQ_10_ supplementation was not administered due to fatal early onset. All these individuals carried homozygous or compound-heterozygous variants, clearly indicating that the disease is inherited as autosomal-recessive trait, indicating that haploinsufficiency might not be pathogenic because the parents, heterozygous for the nonsense variant, were unaffected^[33]^.

Chung *et al.*^[34]^ described five recessive missense mutations in *COQ4* segregating with disease in four families. All patients presented with a severe multisystemic neonatal form including nervous system manifestations such as hypotonia, encephalopathy with EEG abnormalities, neonatal seizures, and cerebellar atrophy. Other manifestations included lactic acidosis, cardiomyopathy, and secondary breathing difficulties. Cerebellar hypoplasia was a common finding and nephropathy was not present. Only two patients received CoQ_10_ supplementation, without response.

Sondheimer *et al.*^[35]^ identified novel mutations in *COQ4* in an infant presenting with early onset biventricular hypertrophic cardiomyopathy, hypotonia, hearing loss, seizures, and lactic acidosis associated with severe muscle CoQ_10_ deficiency.

Ling *et al.*^[36]^ showed three unrelated Chinese families presenting with the *COQ4* c.370G>A (p.G124S) variant, manifesting as either encephalopathy with intractable seizures and developmental delay or cardiomyopathy with left ventricle hypertrophy. In the first case of this series, CoQ_10_ supplementation (600 mg/day) was started at six years, which resulted in improvement in the patient’s alertness only. In the second patient, CoQ_10_ supplementation was started at 250 mg per day; then, it was increased to 400 mg per day, 3 months after symptom onset with some improvement in the control of seizures and patient’s alertness. The patient had only one further episode of epilepsy at the age of three. The third patient was not treated. The same homozygous c.370G>A (p.G124S) *COQ4* variant was reported in another Chinese patient, who presented in the second month of life with Leigh syndrome, respiratory distress, lactic acidosis, dystonia, seizures, and failure to thrive, without renal involvement^[37]^.

A recent paper reported 11 additional southern Chinese patients, the largest cohort of *COQ4* deficient patients to date. Five had classical neonatal-onset encephalo-cardiomyopathy, while the other six had infantile-onset characterized by different constellations of symptoms such as hypotonia, cortical visual impairment, severe developmental delay, and seizures. Although dystonia was observed in two out of the six patients with infantile-onset presentation, none displayed basal ganglia lesions. The patients carried the variant c.370G>A, (p.Gly124Ser), previously reported by Ling *et al.*^[36]^ and Lu *et al.*^[37]^, suggesting a founder effect in the southern Chinese population. Among the 10 patients who received CoQ_10_ supplement and with continuous follow-up, only 3 showed stabilization of the cardiopathy or seizure control; all were homozygous for c.370G>A, p. (Gly124Ser). Some improvement was observed in one patient with the heterozygous missense variants c.370G>A and c.371G>T. Five patients harbored the splicing mutation c.402+1G>A, inducing a severe early onset phenotype that was not responsive to CoQ_10_ supplementation^[38]^.

A recent report expanded the spectrum phenotype of *COQ4* mutations to include childhood-onset spinocerebellar ataxia with stroke-like episodes, associated with a homozygous variant in the *COQ4* gene c.230C>T (p.Thr77Ile), reported in two siblings. After the diagnosis at ages 11 and 13 years, CoQ_10_ supplementation (1000 mg/day) was initiated for both siblings. Although motor outcomes were stable for the first year of treatment, one of the patients developed a second stroke-like episode at age 14^[39]^.

Finally, a homozygous mutation c.164G>T, p.Gly55Val in *COQ4* was reported in two siblings with a combination of slowly progressive ataxia, spasticity, and seizures, constituting an autosomal recessive cerebellar ataxia (ARCA) syndrome. The more severely affected patient received high-dose CoQ_10_ (2000 mg/day) and showed clinically significant improvement; he was originally wheelchair-bound, unable to walk with support or standing unaided. With treatment, he became able to ambulate with a walker and stand without support. After this response, the other patient was also treated, with some improvement as well^[40]^.

### *COQ5* (MIM616359)

*COQ5* catalyzes the only C-methylation in the biosynthesis of CoQ_10_ [Figure 1]^[41]^. Mutations in *COQ5* have been reported in only three sisters of non-consanguineous Iraqi-Jewish descent. They had varying degrees of cerebellar ataxia, encephalopathy, generalized tonic-clonic seizures, and cognitive disability, with childhood onset and slow progression [Table 1 and Figure 2]. Neither WES nor WGS was able to identify a potential pathogenic variant, whereas a SNP array study, performed on the parents and all siblings, identified a tandem duplication affecting the last four exons of the gene, confirmed by Sanger analysis^[42]^.

### *COQ7* (MIM616733)

*COQ7* is required for one of the three hydroxylations of CoQ benzoquinone ring [Figure 1]^[43]^. In 2015, Freyer *et al.*^[44]^ described a 9-year-old boy with *COQ7* pathogenic variants with complex clinical multiple organ involvement. The child had a history of neonatal lung hypoplasia, joint contractures, early infantile hypertension, and left ventricular cardiac hypertrophy, likely secondary to his prenatal kidney dysplasia with renal dysfunction resulting in oligohydramniosis. Although renal dysfunction normalized during the first year of life, he progressively developed mental retardation, axono-demyelinating neuropathy, hypotonia, and hearing loss. The homozygous c.422T>A (p.Val141Glu) variant in *COQ7* was identified through WES. Additional functional studies in the patient fibroblasts confirmed the pathogenicity of the variant.

A second report described a patient carrying the combination of a novel homozygous mutation (p.Leu111Pro) in *COQ7*, with the mitochondrial DNA m.1555A>G mutation, commonly associated with deafness. The phenotype was characterized by a mild form of spastic paraparesia and cognitive impairment as well as hearing loss. No functional studies were performed to define the cause of the deafness. The authors hypothesized that the combination of CoQ_10_ deficiency and the m.1555A>G mutation leads to synergistic inhibition of mitochondrial function, causing irreversible damages and/or cell death and finally the clinical manifestation of hearing loss^[45]^.

In 2019, Kwong *et al.*^[46]^ reported a patient with a severe phenotype characterized by encephalomyonephrocardiopathy, persistent lactic acidosis, and basal ganglia lesions, who died at 12 months. The patient had intrauterine growth restriction, cardiomegaly, and tricuspid regurgitation since antenatal period. WES identified two compound heterozygous variants in the *COQ7* gene: a deletion insertion resulting in frameshift c.599_600delinsTAATGCATC, p.(Lys200Ilefs*56) and a missense substitution c.319C>T, p.(Arg107Trp). The proband started CoQ_10_ supplementation at 2 months of life; the initial dose is unknown, but it was increased to 20 mg/kg/day at 12 months of life. Nevertheless, the patient cardiorespiratory manifestations deteriorated and the patient died of sepsis. Skin fibroblast studies supported pathogenicity by revealing decreased combined complex II + III activity and reduction in CoQ_10_ level.

### *COQ9* (MIM614654)

*COQ9* is required for the stability and function of *COQ7* [Figure 1]^[47,48]^. Mutations in *COQ9* have been reported in few patients, presenting with the similar lethal neonatal phenotypes characterized by encephalomyopathy and kidney involvement, including tubulopathy [Table 1 and Figure 2].

In 2009, Duncan *et al.*^[49]^ described the first variant in *COQ9* (c.730C>T, p.Arg244*), in a patient from an apparently non-consanguineous Pakistani family, who presented with neonatal lactic acidosis, intractable seizures, global developmental delay, microcephaly, dystonia, left ventricular hypertrophy, and renal tubular dysfunction^[50]^.

Danhauser *et al.*^[51]^ described another infant carrying a homozygous splice-site variant c.521+1del, p.(Ser127_Arg202del) in *COQ9*, manifesting with neonatal encephalopathy with hypotonia, poor breathing, and severe lactic acidosis with symmetrical hyperechoic signal alterations in the basal ganglia, suggestive of neonatal Leigh-like syndrome. The patient subsequently developed seizures and recurrent episodes of apnea and bradycardia and died at 18 days of life.

In 2018, Smith *et al.*^[52]^ reported four siblings, who presented prenatally with an unknown and an ultimately lethal condition characterized by intrauterine growth retardation, oligohydramnios, variable dilated cardiomyopathy, anemia, abnormal appearing kidneys, and autopsy brain findings suggestive of Leigh disease. The patients had the variants c.521+2T>C and c.711+3G>C in *COQ9*, which cause in-frame deletions (p.Ser127_Arg202del and p. Ala203_Asp237del).

In 2019, a novel frameshift c.384delG (Gly129Valfs*17) homozygous mutation was reported in a 9-month-old girl, born from consanguineous parents of Pakistani origin, presenting with growth retardation, microcephaly, and seizures. She was born at 38 weeks gestation, weighed 2000 g, after an uncomplicated pregnancy, and was hospitalized for 3 days due to respiratory distress. At age 4 months, she had sustained clonic seizures. Physical examination showed microcephaly, truncal hypotonia, and dysmorphic features. Abdominal ultrasonography revealed cystic kidneys. Non-compaction of the left ventricle was detected in echocardiography. Cranial MRI showed hypoplasia of the cerebellar vermis and brain stem, corpus callosum agenesis, and cortical atrophy. CoQ_10_ supplementation (5 mg/kg/day) was started when she was 10 months old. Despite increasing the dose to 50 mg/kg/day after the molecular diagnosis, no neurological improvement was observed^[53]^.

## DIAGNOSIS OF EARLY ONSET MULTISYSTEMIC PHENOTYPE OF PRIMARY COQ_10_ DEFICIENCY

Early onset primary CoQ_10_ deficiency is clinically heterogeneous, and genotype-phenotype correlation is based on a limited number of cases^[9,10]^. Four phenotypic groups can be defined: (1) SRNS, isolated or with neurological involvement, associated with defects in *PDSS2, COQ2, COQ6*, or *COQ8B* (the latter with later age-at-onset); (2) encephalomyopathy, hypertrophic/dilated cardiomyopathy, lactic acidosis, and tubulopathy with defects in *PDSS2*, *COQ2, COQ7*, or *COQ9*; (3) neonatal cardio-encephalopathies with *COQ2, COQ4*, or *PDSS1*; and (4) pure neurological syndromes, including isolated or combined Leigh syndrome, ARCA, and refractory epilepsy, in association with defects in *COQ2, COQ4, COQ5, COQ7*, or *COQ9* [Table 1 and Figure 2]^[9,10]^.

In general, clinical features alone are insufficient to definitively diagnose CoQ_10_ deficiency or to distinguish between primary and secondary CoQ_10_ deficiencies, or even from other mitochondrial conditions. Therefore, evaluation of patients with suspected CoQ_10_ deficiency relies on genetic or biochemical studies. If the clinical picture and/or family history raise the possibility of a metabolic/genetic condition, WES, including sequencing of mitochondrial DNA, if available, should be considered the first step. However, only 35% of Mendelian diseases are solved by WES^[54]^ because the majority of undiagnosed cases are subject to limitations in variant-calling and prioritization, as well as inability to detect intronic and regulatory pathogenic variants. WGS enables complete coverage of the genome; however, interpretation is often hindered by difficulty in prioritization of the vast numbers of variants detected and our incomplete understanding of the non-coding sequences. Consequently, the diagnostic yield with WGS is only modestly increased to just over 40%^[55–57]^. In parallel with NGS, laboratory analyses should include routine tests such as blood lactate and urine organic acids, although normal values do not exclude CoQ_10_ deficiency.

If genetic analysis shows pathogenic homozygous or compound heterozygous variants in any of the previously reported genes involved in CoQ_10_ synthesis with a compatible clinical picture, definitive diagnosis of primary CoQ_10_ can be established without further analyses. In presence of variants of uncertain significance, functional and/or complementary studies are needed. Blood mononuclear cells represent a readily accessible sample, which is often suitable as an alternative to muscle for the measurement of CoQ_10_, by high performance liquid chromatography or mass spectrometry^[11,58]^. In contrast, plasma levels of CoQ_10_ are influenced by the amount of plasma lipoproteins (carriers of CoQ_10_ in circulation), dietary intake, or supplementation, therefore cannot be used for diagnostic purpose. In addition, COQ_10_ levels can be measured in other tissues, such as lymphoblastoid cell lines or primary fibroblasts, although normal values in these tissues do not exclude the diagnosis of CoQ_10_ deficiency, as some patients with genetically confirmed CoQ_10_ biosynthetic defects have had normal CoQ_10_ levels in fibroblasts. As mentioned above, reduced activity of complexes I + III and II + III (and I + III) is highly suggestive of CoQ_10_ deficiency^[10]^.

## TREATMENT OF EARLY ONSET MULTISYSTEMIC PHENOTYPE OF PRIMARY COQ_10_ DEFICIENCY

### Current treatments

#### Humans

Varying doses of CoQ_10_ have been used for the treatment of primary CoQ_10_ deficiencies, ranging from 5 to 50 mg/kg/day for both adults and children^[10,17]^. We cannot compare the effects of different dosages because formulations and durations of treatment also varied^[10]^. We recommend high doses of CoQ_10_ supplementation (> 30 mg/kg), because inadequate dosage and duration of intake have often constrained uptake of exogenous CoQ_10_^[59–61]^, with few mild reported side effects^[10]^.

Early intervention with CoQ_10_ supplementation in high doses has been shown to improve renal function^[62]^. However, in neonatal cases with neurological involvement, response of CoQ_10_ supplementation is poor, probably due to the irreversible brain damage at the time of the diagnosis, as well as the poor bioavailability of CoQ_10_, which does not cross the blood-brain barrier^[29,46,53]^. New solubilized and stabilized formulations that are able to preserve CoQ_10_ in its reduced form (CoQH_2_ or ubiquinol) have been developed and increase bioavailability after oral dosing compared to standard ubiquinone^[63]^. Experience in patients with primary CoQ_10_ deficiency is limited and there are no clear indications about the dose-equivalence of ubiquinone and ubiquinol. Short-tail Q_10_ analogs, such as idebenone (IDB), are more bioavailable than CoQ_10_ but are not effective in patients with primary CoQ_10_ deficiency^[64]^.

#### In vitro and in vivo studies

*In vitro* studies in human fibroblasts show that short-tail Q_10_ analogs, such as CoQ_2_ and IDB, are not effective in primary CoQ_10_ deficiency because they do not correct the respiratory chain defects^[65]^.

Studies in *Pdss2* mutant mice, a mouse model of CoQ-deficient NS, show that CoQ_10_ supplementation prevents renal failure through rescue of sulfides metabolism and oxidative stress. In contrast, IDB treatment was ineffective and comparable to placebo^[66,67]^.

In a mouse model of CoQ_10_ deficiency and encephalomyopathy due to *Coq9* dysfunction, the water-soluble formulation of ubiquinol was shown to be more effective than ubiquinone in rescuing brain abnormalities^[68]^.

### Investigational treatments

Administration of metabolic intermediates able to “bypass” the enzymatic block and to enable endogenous synthesis of CoQ_10_ has been attempted in experimental *in vitro* and *in vivo* models of primary CoQ deficiency, as an alternative to CoQ_10_ supplementation^[69]^, whose therapeutic effects are hampered by its poor bioavailability.

#### In vitro studies

Treatment with 2,4-dihydroxybenzoic acid (DHB, β-resorcylic acid, β-RA) was shown to be effective in human fibroblasts carrying *COQ7* pathogenic variants^[44,45]^ and in COQ2-deficient cell lines, increasing the levels of CoQ_10_ as well as increasing the viability of mutant cells growth in galactose medium^[70]^.

Luna-Sánchez *et al.*^[71]^ also investigated the effect of DHB in mouse embryonic fibroblasts from two different mouse models of COQ9 dysfunction (*Coq9^R239X/R239X^* and *Coq9^Q95X/Q95X^*) showing similar results to those obtained in COQ2 and COQ7 mutant cells, with different response to treatment based on the severity of the biochemical defect and the residual levels of *COQ7*.

Treatment with vanillic acid (VA) recovered CoQ_10_ biosynthesis, ATP production, and reduced levels of reactive oxygen species in a human cell line lacking functional *COQ6*^[72]^, a FAD-dependent monooxygenase responsible for the addition of the hydroxyl group in position C5 of the quinone ring^[73]^. Mutations in *COQ6* cause SRNS associated with sensorineural deafness and a variable degree of encephalopathy^[74]^.

#### In vivo studies

The first studies to show *in vivo* efficacy of hydroxylated CoQ precursor compounds 3,4-dihydroxybenzoic acid, DHB, and VA to rescue endogenous CoQ biosynthesis were performed in yeast models of *COQ6* and *COQ7* deficiencies^[75,76]^.

More recent studies showed that DHB ameliorated survival and phenotype in *Coq7* knock-out mice^[77]^, while vanillic acid ameliorated proteinuria and prevented focal segmental glomerulosclerosis in podocyte-specific *Coq6* knockout mice (*Coq6^podKO^*), prolonging their survival^[78]^.

DHB was found to rescue not only the clinical phenotype but also morphological and histopathological signs of encephalopathy in the *Coq9^R239X^* mouse. The therapeutic effect of DHB was not attributed to the increase of CoQ_10_ levels, but rather to the reduction of DMQ_10_, an intermediated metabolite that may be toxic for mitochondrial function when accumulated in the organelle. Thus, the authors proposed that DHB should be preferentially considered for the treatment of human CoQ_10_ deficiency with accumulation of DMQ_10_, as mutations in COQ4, COQ7, and *COQ9*^[79]^.

Although all these experimental data suggest that biosynthesis intermediates might be a promising alternative, further studies are needed to assess therapeutic response, safety, and bioavailability and to understand their mechanism of action before their translation to the clinical practice.

## CONCLUSION

Multisystemic forms of primary CoQ_10_ deficiency are usually devastating conditions manifesting in prenatal, neonatal, or infantile period of life. Clinical symptoms include variable combinations of encephalomyocardionephropathy syndromes. Although the diagnosis of these primary CoQ_10_ deficiency syndromes is usually not straightforward, renal involvement, particularly SRNS, can be a clinical clue. In the severe multisystemic forms, WES is often the first step in the diagnostic workup. Nevertheless, detection of novel genetic variants of uncertain significance should be followed by biochemical assays and/or functional studies in patient cells to prove pathogenicity. Eventually, comprehensive characterization of the clinical spectrum of these syndromes and associated molecular defects will establish pathogenicity of variants identified by WES and obviate further studies that are available only in specialized research laboratories.

Although in the suspect of primary coenzyme Q_10_ deficiency high doses of coenzyme Q_10_ supplementation are recommended, early-onset neurological features are often not responsive to supplementation. CoQ_10_ biosynthetic analogs might be suitable alternatives to CoQ_10_ supplementation, but additional analyses are required before these compounds can be translated to the clinical setting.

## Figures and Tables

**Table 1.** Clinical features associated with specific defects in CoQ biosynthesis

Gene	Clinical features
*PDSS1* (MIM607429)	Deafness, encephaloneuropathy, obesity, livedo reticularis, cardiopathy developmental delay, nephrotic syndrome, failure to thrive, Leigh syndrome
*PDSS2* (MIM610564)	SRNS, neurological involvement (ataxia, dystonia, amyotrophia), Leigh syndrome, retinitis pigmentosa, sensorineural deafness, cardiopathy, lactic acidosis, failure to thrive, optic atrophy
*COQ2* (MIM609825)	SRNS, encephalopathy (including stroke-like episodes), Multiple-System Atrophy, retinopathy, seizures, hypotonia, psychomotor delay, nystagmus and optic atrophy
*COQ4* (MIM 616227)	Cardiopathy, encephalomyopathy, hypotonia, cortical visual impairment, severe developmental delay, seizures
*COQ5* (MIM616359)	Cerebellar ataxia, encephalopathy, generalized tonic-clonic seizures, cognitive disability
*COQ7* (MIM616733)	Cardiopathy, neonatal lung hypoplasia, contractures, renal dysfunction (including kidney dysplasia), spastic paraplegia, cognitive impairment, deafness, encephalopathy, Leigh syndrome, lactic acidosis
*COQ9* (MIM614654)	Cardiopathy, lactic acidosis, seizures, developmental delay, microcephaly, dystonia, renal tubular dysfunction, Leigh-like syndrome

SRNS: steroid resistant nephrotic syndrome

## References

[1] Turunen M, Olsson J, Dallner G. Metabolism and function of coenzyme Q. Biochim Biophys Acta 2004;1660:171–99. 14757233 10.1016/j.bbamem.2003.11.012

[2] Rebrin I, Kamzalov S, Sohal RS. Tissue bioavailability and detection of coenzyme Q. Methods Enzymol 2004;378:138–45. 15038963 10.1016/S0076-6879(04)78009-X

[3] Kaurola P, Sharma V, Vonk A, Vattulainen I, Róg T. Distribution and dynamics of quinones in the lipid bilayer mimicking the inner membrane of mitochondria. Biochim Biophys Acta 2016;1858:2116–22. 27342376 10.1016/j.bbamem.2016.06.016

[4] Díaz-Casado ME, Quiles JL, Barriocanal-Casado E, González-García P, Battino M, The paradox of coenzyme Q 10 in aging. Nutrients 2019;11:2221. 10.3390/nu11092221 PMC6770889 31540029

[5] Kawamukai M Biosynthesis of coenzyme Q in eukaryotes. Biosci Biotechnol Biochem 2015;80:23–33. 26183239 10.1080/09168451.2015.1065172

[6] Xia L, Nordman T, Olsson JM, Damdimopoulos A, Björkhem-Bergman L, The mammalian cytosolic selenoenzyme thioredoxin reductase reduces ubiquinone. A novel mechanism for defense against oxidative stress. J Biol Chem 2003;278:2141–6. 12435734 10.1074/jbc.M210456200

[7] Vadhanavikit S, Ganther HE. Decreased ubiquinone levels in tissues of rats deficient in selenium. Biochem Biophys Res Commun 1993;190:921–6. 8439341 10.1006/bbrc.1993.1137

[8] Vadhanavikit S, Ganther HE. Selenium deficiency and decreased coenzyme Q levels. Mol Aspects Med 1994;15:s103–7. 7752821 10.1016/0098-2997(94)90019-1

[9] Alcázar-Fabra M, Trevisson E, Brea-Calvo G. Clinical syndromes associated with Coenzyme Q10 deficiency. Essays Biochem 2018;62:377–98. 30030365 10.1042/EBC20170107

[10] Emmanuele V, López LC, Berardo A, Naini A, Tadesse S, Heterogeneity of coenzyme Q10 deficiency: patient study and literature review. Arch Neurol 2012;69:978–83. 22490322 10.1001/archneurol.2012.206 PMC3639472

[11] Barca E, Kleiner G, Tang G, Ziosi M, Tadesse S, Decreased coenzyme Q10 levels in multiple system atrophy cerebellum. J Neuropathol Exp Neurol 2016;75:663–72. 27235405 10.1093/jnen/nlw037 PMC4913434

[12] Lagier-Tourenne C, Tazir M, Lopez LC, Quinzii CM, Assoum M, ADCK3, an ancestral kinase, is mutated in a form of recessive ataxia associated with coenzyme Q10 deficiency. Am J Hum Genet 2008;82:661–72. 18319074 10.1016/j.ajhg.2007.12.024 PMC2427193

[13] Mollet J, Delahodde A, Serre V, Chretien D, Schlemmer D, CABC1 gene mutations cause ubiquinone deficiency with cerebellar ataxia and seizures. Am J Hum Genet 2008;82:623–30. 18319072 10.1016/j.ajhg.2007.12.022 PMC2427298

[14] Dinwiddie DL, Smith LD, Miller NA, Atherton AM, Farrow EG, Diagnosis of mitochondrial disorders by concomitant next-generation sequencing of the exome and mitochondrial genome. Genomics 2013;102:148–56. 23631824 10.1016/j.ygeno.2013.04.013 PMC4557607

[15] Mollet J, Giurgea I, Schlemmer D, Dallner G, Chretien D, Prenyldiphosphate synthase, subunit 1 (PDSS1) and OH-benzoate polyprenyltransferase (COQ2) mutations in ubiquinone deficiency and oxidative phosphorylation disorders. J Clin Invest 2007;117:765–72. 17332895 10.1172/JCI29089 PMC1804361

[16] Vasta V, Merritt JL, Saneto RP, Hahn SH. Next-generation sequencing for mitochondrial diseases: a wide diagnostic spectrum. Pediatr Int 2012;54:585–601. 22494076 10.1111/j.1442-200X.2012.03644.x

[17] Rötig A, Appelkvist EL, Geromel V, Chretien D, Kadhom N, Quinone-responsive multiple respiratory-chain dysfunction due to widespread coenzyme Q10 deficiency. Lancet 2000;356:391–95. 10972372 10.1016/S0140-6736(00)02531-9

[18] López LC, Schuelke M, Quinzii CM, Kanki T, Rodenburg RJT, Leigh syndrome with nephropathy and CoQ10 deficiency due to decaprenyl diphosphate synthase subunit 2 (PDSS2) mutations. Am J Hum Genet 2006;79:1125–29. 17186472 10.1086/510023 PMC1698707

[19] Quinzii C, Loos M. Multisystemic infantile CoQ10 deficiency with renal involvement In: Saneto RP, Parikh S, Cohen BH, editors. Mitochondrial case studies underlying mechanisms and diagnosis. Academic Press; 2016 pp. 299–304.

[20] Iványi B, Rácz GZ, Gál P, Brinyiczki K, Bódi I, Diffuse mesangial sclerosis in a PDSS2 mutation-induced coenzyme Q10 deficiency. Pediatr Nephrol 2018;33:439–46. 29032433 10.1007/s00467-017-3814-1

[21] Sadowski CE, Lovric S, Ashraf S, Pabst WL, Gee HY, A single-gene cause in 29.5% of cases of steroid-resistant nephrotic syndrome. J Am Soc Nephrol 2015;26:1279–89. 25349199 10.1681/ASN.2014050489 PMC4446877

[22] Forsgren M, Attersand A, Lake S, Grünler J, Swiezewska E, Isolation and functional expression of human COQ2, a gene encoding a polyprenyl transferase involved in the synthesis of CoQ. Biochem J 2004;382:519–26. 15153069 10.1042/BJ20040261 PMC1133808

[23] Salviati L, Sacconi S, Murer L, Zacchello G, Franceschini L, Infantile encephalomyopathy and nephropathy with CoQ10 deficiency: a CoQ10-responsive condition. Neurology 2005;65:606–8. 16116126 10.1212/01.wnl.0000172859.55579.a7

[24] Quinzii C, Naini A, Salviati L, Trevisson E, Navas P, A mutation in para-hydroxybenzoate-polyprenyl transferase (COQ2) causes primary coenzyme Q10 deficiency. Am J Hum Genet 2006;78:345–49. 16400613 10.1086/500092 PMC1380241

[25] Desbats MA, Vetro A, Limongelli I, Lunardi G, Casarin A, Primary coenzyme Q10 deficiency presenting as fatal neonatal multiorgan failure. Eur J Hum Genet 2015;23:1254–58. 25564041 10.1038/ejhg.2014.277 PMC4430297

[26] Jakobs BS, van den Heuvel LP, Smeets RJP, de Vries MC, Hien S, A novel mutation in COQ2 leading to fatal infantile multisystem disease. J Neurol Sci 2013;326:24–8. 23343605 10.1016/j.jns.2013.01.004

[27] Scalais E, Chafai R, Van Coster R, Bindl L, Nuttin C, Early myoclonic epilepsy, hypertrophic cardiomyopathy and subsequently a nephrotic syndrome in a patient with CoQ10 deficiency caused by mutations in para-hydroxybenzoate-polyprenyl transferase (COQ2). Eur J Paediatr Neurol 2013;17:625–30. 23816342 10.1016/j.ejpn.2013.05.013

[28] Diomedi-Camassei F, Di Giandomenico S, Santorelli FM, Caridi G, Piemonte F, COQ2 nephropathy: a newly described inherited mitochondriopathy with primary renal involvement. J Am Soc Nephrol 2007;18:2773–80. 17855635 10.1681/ASN.2006080833

[29] Eroglu F, Ozaltin F, Gönç N, Nalçacıoğlu H, Birsin Özçakar Z, Response to early coenzyme Q10 supplementation is not sustained in CoQ10 deficiency caused by CoQ2 mutation. Pediatr Neurol 2018;88:71–4. 30337132 10.1016/j.pediatrneurol.2018.07.008

[30] Mitsui J, Matsukawa T, Ishiura H, Fukuda Y, Ichikawa Y, Mutations in COQ2 in familial and sporadic multiple-system atrophy. N Engl J Med 2013;369:233–44. 23758206 10.1056/NEJMoa1212115

[31] Belogrudov GI, Lee PT, Jonassen T, Hsu AY, Gin P, Yeast COQ4 encodes a mitochondrial protein required for coenzyme Q synthesis. Arch Biochem Biophys 2001;392:48–58. 11469793 10.1006/abbi.2001.2448

[32] Salviati L, Trevisson E, Rodriguez Hernandez MA, Casarin A, Pertegato V, Haploinsufficiency of COQ4 causes coenzyme Q10 deficiency. J Med Genet 2012;49:187–91. 22368301 10.1136/jmedgenet-2011-100394 PMC3983946

[33] Brea-Calvo G, Haack TB, Karall D, Ohtake A, Invernizzi F, COQ4 mutations cause a broad spectrum of mitochondrial disorders associated with CoQ10 deficiency. Am J Hum Genet 2015;96:309–17. 25658047 10.1016/j.ajhg.2014.12.023 PMC4320255

[34] Chung WK, Martin K, Jalas C, Braddock SR, Juusola J, Mutations in COQ4, an essential component of coenzyme Q biosynthesis, cause lethal neonatal mitochondrial encephalomyopathy. J Med Genet 2015;52:627–35. 26185144 10.1136/jmedgenet-2015-103140

[35] Sondheimer N, Hewson S, Cameron JM, Somers GR, Broadbent JD, Novel recessive mutations in COQ4 cause severe infantile cardiomyopathy and encephalopathy associated with CoQ 10 deficiency. Mol Genet Metab Rep 2017;12:23–7. 28540186 10.1016/j.ymgmr.2017.05.001 PMC5432661

[36] Ling TK, Law CY, Yan KW, Fong NC, Wong KC, Clinical whole-exome sequencing reveals a common pathogenic variant in patients with CoQ10 deficiency: An underdiagnosed cause of mitochondriopathy. Clin Chim Acta 2019;497:88–94. 31325447 10.1016/j.cca.2019.07.016

[37] Lu M, Zhou Y, Wang Z, Xia Z, Ren J, Clinical phenotype, in silico and biomedical analyses, and intervention for an east asian population-specific c.370G>A (p.G124S) COQ4 mutation in a chinese family with CoQ10 deficiency-associated leigh syndrome. J Hum Genet 2019;64:297–304. 30659264 10.1038/s10038-019-0563-y

[38] Yu MH, Tsang MH, Lai S, Ho MS, Tse DML, Primary coenzyme Q10 deficiency-7: expanded phenotypic spectrum and a founder mutation in Southern Chinese. NPJ Genom Med 2019;4:18. 31396399 10.1038/s41525-019-0091-x PMC6683205

[39] Bosch AM, Kamsteeg EJ, Rodenburg RJ, van Deutekom AW, Buis DR, Coenzyme Q10 deficiency due to a COQ4 gene defect causes childhood-onset spinocerebellar ataxia and stroke-like episodes. Mol Genet Metab Rep 2018;17:19–21. 30225196 10.1016/j.ymgmr.2018.09.002 PMC6138878

[40] Caglayan AO, Gumus H, Sandford E, Kubisiak TL, Ma Q, COQ4 mutation leads to childhood-onset ataxia improved by CoQ10 administration. Cerebellum 2019;18:665. 30847826 10.1007/s12311-019-01011-x PMC6536000

[41] Nguyen T, Casarin A, Desbats MA, Doimo M, Trevisson E, Molecular characterization of the human COQ5 C-methyltransferase in coenzyme Q10 biosynthesis. Biochim Biophys Acta 2014;184:1628–38. 10.1016/j.bbalip.2014.08.007 PMC4331671 25152161

[42] Malicdan MC, Vilboux T, Ben-Zeev B, Guo J, Eliyahu A, A novel inborn error of the coenzyme Q10 biosynthesis pathway: cerebellar ataxia and static encephalomyopathy due to COQ5 C-methyltransferase deficiency. Hum Mutat 2018;39:69–79. 29044765 10.1002/humu.23345 PMC5722658

[43] Stenmark P, Grünler J, Mattsson J, Sindelar PJ, Nordlund P, A new member of the family of di-iron carboxylate proteins. Coq7 (clk-1), a membrane-bound hydroxylase involved in ubiquinone biosynthesis. J Biol Chem 2001;276:33297–300. 11435415 10.1074/jbc.C100346200

[44] Freyer C, Stranneheim H, Naess K, Mourier A, Felser A, Rescue of primary ubiquinone deficiency due to a novel COQ7 defect using 2,4-dihydroxybensoic acid. J Med Genet 2015;52:779–83. 26084283 10.1136/jmedgenet-2015-102986 PMC4680133

[45] Wang Y, Smith C, Parboosingh JS, Khan A, Innes M, Pathogenicity of two COQ7 mutations and responses to 2,4-dihydroxybenzoate bypass treatment. J Cell Mol Med 2017;21:2329–43. 28409910 10.1111/jcmm.13154 PMC5618687

[46] Kwong A, Chiu A, Tsang M, Lun K, Richard J, A fatal case of COQ7-associated primary coenzyme Q 10 deficiency. JIMD Rep 2019;47:23–9. 31240163 10.1002/jmd2.12032 PMC6498831

[47] García-Corzo L, Luna-Sánchez M, Doerrier C, García JA, Guarás A, Dysfunctional Coq9 protein causes predominant encephalomyopathy associated with CoQ deficiency. Hum Mol Genet 2013;22:1233–48. 23255162 10.1093/hmg/dds530

[48] Lohman DC, Forouhar F, Beebe ET, Stefely MS, Minogue CE, Mitochondrial COQ9 is a lipid-binding protein that associates with COQ7 to enable coenzyme Q biosynthesis. Proc Natl Acad Sci U S A 2014;111:E4697–705. 25339443 10.1073/pnas.1413128111 PMC4226113

[49] Duncan AJ, Bitner-Glindzicz M, Meunier B, Costello H, Hargreaves IP, A nonsense mutation in COQ9 causes autosomal-recessive neonatal-onset primary coenzyme Q10 deficiency: a potentially treatable form of mitochondrial disease. Am J Hum Genet 2009;84:558–66. 19375058 10.1016/j.ajhg.2009.03.018 PMC2681001

[50] Rahman S, Hargreaves I, Clayton P, Heales S. Neonatal presentation of coenzyme Q10 deficiency. J Pediatr 2001;139:456–8. 11562630 10.1067/mpd.2001.117575

[51] Danhauser K, Herebian D, Haack TB, Rodenburg RJ, Strom TM, Fatal neonatal encephalopathy and lactic acidosis caused by a homozygous loss-of-function variant in COQ9. Eur J Hum Genet 2016;24:450–4. 26081641 10.1038/ejhg.2015.133 PMC4755375

[52] Smith AC, Ito Y, Ahmed A, Schwartzentruber JA, Beaulieu CL, A family segregating lethal neonatal coenzyme Q 10 deficiency caused by mutations in COQ9. J Inherit Metab Dis 2018;41:719–29. 29560582 10.1007/s10545-017-0122-7

[53] Olgac A, Öztoprak Ü, Kasapkara ÇS, Kılıç M, Yüksel D, A rare case of primary coenzyme Q10 deficiency due to COQ9 mutation. J Pediatr Endocrinol Metab 2020;33:165–70. 31821167 10.1515/jpem-2019-0245

[54] Zhang X Exome sequencing greatly expedites the progressive research of mendelian diseases. Front Med 2014;8:42–57. 24384736 10.1007/s11684-014-0303-9

[55] Yang Y, Muzny DM, Xia F, Niu Z, Person R, Molecular findings among patients referred for clinical whole-exome sequencing. JAMA 2014;312:1870–9. 25326635 10.1001/jama.2014.14601 PMC4326249

[56] Taylor JC, Martin HC, Lise S, Broxholme J, Cazier JB, Factors influencing success of clinical genome sequencing across a broad spectrum of disorders. Nat Genet 2015;47:717–26. 25985138 10.1038/ng.3304 PMC4601524

[57] Chong JX, Buckingham KJ, Jhangiani SN, Boehm C, Sobreira N, The genetic basis of Mendelian phenotypes: discoveries, challenges, and opportunities. Am J Human Genet 2015;97:199–215. 26166479 10.1016/j.ajhg.2015.06.009 PMC4573249

[58] Yubero D, Allen G, Artuch R, Montero R. The value of coenzyme q10 determination in mitochondrial patients. J Clin Med 2017;6:37. 10.3390/jcm6040037 PMC5406769 28338638

[59] Bhagavan HN, Chopra RK. Coenzyme Q10: absorption, tissue uptake, metabolism and pharmacokinetics. Free Radic Res 2006;40:445–53. 16551570 10.1080/10715760600617843

[60] Zaki NM. Strategies for oral delivery and mitochondrial targeting of CoQ10. Drug Deliv 2016;23:1868–81. 25544601 10.3109/10717544.2014.993747

[61] Kwong LK, Kamzalov S, Rebrin I, Bayne VAC, Jana CK, Effects of coenzyme Q(10) administration on its tissue concentrations, mitochondrial oxidant generation, and oxidative stress in the rat. Free Radic Biol Med 2002;33:627–38. 12208349 10.1016/s0891-5849(02)00916-4

[62] Montini G, Malaventura C, Salviati L. Early coenzyme Q10 supplementation in primary coenzyme Q10 deficiency. N Engl J Med 2008;358:2849–50. 18579827 10.1056/NEJMc0800582

[63] Bhagavan HN, Chopra RK. Plasma coenzyme Q10 response to oral ingestion of coenzyme Q10 formulations. Mitochondrion 2007;7:S78–88. 17482886 10.1016/j.mito.2007.03.003

[64] Auré K, Benoist JF, Ogier de Baulny H, Romero NB, Rigal O, Progression despite replacement of a myopathic form of coenzyme Q10 defect. Neurology 2004;63:727–9. 15326254 10.1212/01.wnl.0000134607.76780.b2

[65] López LC, Quinzii CM, Area E, Naini A, Rahman S, Treatment of CoQ(10) deficient fibroblasts with ubiquinone, CoQ analogs, and vitamin C: time- and compound-dependent effects. PLoS One 2010;5:e11897. 20689595 10.1371/journal.pone.0011897 PMC2912846

[66] Kleiner G, Barca E, Ziosi M, Emmanuele V, Xu Y, CoQ 10 Supplementation Rescues Nephrotic Syndrome Through Normalization of H 2 S Oxidation Pathway. Biochim Biophys Acta Mol Basis Dis 2018;1864:3708–22. 30251690 10.1016/j.bbadis.2018.09.002 PMC6181133

[67] Saiki R, Lunceford AL, Shi Y, Marbois B, King R, Coenzyme Q10 supplementation rescues renal disease in Pdss2kd/kd mice with mutations in prenyl diphosphate synthase subunit 2. Am J Physiol Renal Physiol 2008;295:F1535–44. 18784258 10.1152/ajprenal.90445.2008 PMC2584909

[68] García-Corzo L, Luna-Sánchez M, Doerrier C, Ortiz F, Escames G, Ubiquinol-10 ameliorates mitochondrial encephalopathy associated with CoQ deficiency. Biochim Biophys Acta 2014;1842:893–901. 24576561 10.1016/j.bbadis.2014.02.008

[69] Herebian D, López LC, Distelmaier F. Bypassing human CoQ10 deficiency. Mol Genet Metab 2018;123:289–91. 29246431 10.1016/j.ymgme.2017.12.008

[70] Herebian D, Seibt A, Smits SHJ, Rodenburg RJ, Mayatepek E, 4-Hydroxybenzoic acid restores CoQ 10 biosynthesis in human COQ2 deficiency. Ann Clin Transl Neurol 2017;4:902–8. 29296619 10.1002/acn3.486 PMC5740244

[71] Luna-Sánchez M, Díaz-Casado E, Barca E, Tejada MA, Montilla-García A, The clinical heterogeneity of coenzyme Q10 deficiency results from genotypic differences in the Coq9 gene. EMBO Mol Med 2015;7:670–87. 25802402 10.15252/emmm.201404632 PMC4492823

[72] Acosta Lopez M, Trevisson E, Canton M, Vazquez-Fonseca L, Morbidoni V, Vanillic acid restores coenzyme Q biosynthesis and ATP production in human cells lacking COQ6. Oxid Med Cell Longev 2019;2019:3904905. 31379988 10.1155/2019/3904905 PMC6652073

[73] Ozeir M, Muhlenhoff U, Webert H, Lill R, Fontecave M, Coenzyme Q biosynthesis: Coq6 is required for the C5-hydroxylation reaction and substrate analogs rescue Coq6 deficiency. Chem Biol 2011;18:1134–42. 21944752 10.1016/j.chembiol.2011.07.008

[74] Park E, Ahn YH, Kang HG, Yoo KH, Won NH, COQ6 mutations in children with steroid-resistant focal segmental glomerulosclerosis and sensorineural hearing loss. Am J Kidney Dis 2017;70:139–44. 28117207 10.1053/j.ajkd.2016.10.040

[75] Doimo M, Trevisson E, Airik R, Bergdoll M, Santos-Ocaña C, Effect of vanillic acid on COQ6 mutants identified in patients with coenzyme Q10 deficiency. Biochim Biophys Acta 2014;1842:1–6. 24140869 10.1016/j.bbadis.2013.10.007 PMC3898990

[76] Xie LX, Ozeir M, Tang JY, Chen JY, Jaquinod SK, Overexpression of the Coq8 kinase in Saccharomyces cerevisiae coq null mutants allows for accumulation of diagnostic intermediates of the coenzyme Q6 biosynthetic pathway. J Biol Chem 2012;287:23571–81. 22593570 10.1074/jbc.M112.360354 PMC3390632

[77] Wang Y, Oxer D, Hekimi S. Mitochondrial function and lifespan of mice with controlled ubiquinone biosynthesis. Nat Commun 2015;6:6393. 25744659 10.1038/ncomms7393 PMC4715896

[78] Widmeier E, Airik M, Hannah H, Schapiro D, Wedel J, Treatment with 2,4-dihydroxybenzoic acid prevents FSGS progression and renal fibrosis in podocyte-specific Coq6 knockout mice. JASN 3 2019;30:393–405. 10.1681/ASN.2018060625 PMC6405149 30737270

[79] Hidalgo-Gutiérrez A, Barriocanal-Casado E, Bakkali M, Díaz-Casado ME, Sánchez-Maldonado L, β-RA reduces DMQ/CoQ ratio and rescues the encephalopathic phenotype in Coq9 R239X mice. EMBO Mol Med 2019;11:e9466. 30482867 10.15252/emmm.201809466 PMC6328940

